# Outpatient parenteral antimicrobial therapy (OPAT) for aortic vascular graft infection; a five-year retrospective evaluation

**DOI:** 10.1186/s12879-021-06373-4

**Published:** 2021-07-09

**Authors:** Niamh Allen, Mohamed Adam, Grace O’Regan, Aoife Seery, Cora McNally, Samuel McConkey, Aisling Brown, Eoghan de Barra

**Affiliations:** 1 Department of Infectious Diseases, Beaumont Hospital, Dublin, Ireland; 2 Department of International Health and Tropical Medicine, Royal College of Surgeons of Ireland (RSCI), University of Medicine and Health Sciences, Dublin, Ireland; 3 Department of Infectious Diseases, Guy’s and St. Thomas’ NHS Foundation Trust, London, UK

**Keywords:** Aortic graft infection (AGI), Endovascular aortic aneurysm repair (EVAR), Vascular graft infection (VGI), Outpatient parenteral antimicrobial therapy (OPAT), Oral suppressive antimicrobial therapy

## Abstract

**Objectives:**

An estimated 1% of endovascular aneurysm repair (EVAR) devices become infected, carrying a high mortality rate. Surgical explantation is recommended and prognosis is guarded. This retrospective cohort analysis focuses on the role of outpatient parenteral antimicrobial therapy (OPAT) in the management of aortic vascular graft infections following EVAR.

**Methods:**

Patients who received OPAT for aortic graft infections (AGI) following EVAR from 2014 to 2018 inclusive were identified using the OPAT database. Clinical, microbiological and radiological data were collected. Survivors were followed up for a median of 36 months (range 25–60) after first presentation with infection. Outcomes were assessed.

**Results:**

Eleven cases with 20 OPAT episodes were identified: 10/11 male, median age 76 (IQR 71–81). Median time to presentation was 7 months (range 0–81 months) after EVAR. OPAT lead to a 55% reduction in length of hospital stay. One patient had graft explantation; four others had temporising measures. Eight of 11 were alive a median of 36 months after presentation with infection, having had a median of 2 re-treatments on OPAT (range 1–3). Seven of the eight survivors were on continuous suppressive oral antimicrobials; three were also intermittently on intravenous antibiotics for flares of infection. Patient/ infection outcomes were cure (1/11), improved (7/11), failure (3/11).

**Conclusion:**

AGI following EVAR usually presents in the first year after graft deployment. OPAT has an important peri-operative role in patients suitable for curative surgery. OPAT followed by oral suppressive antimicrobial therapy can be a feasible long-term treatment for non-curative management of AGI. Survival in our cohort was longer than expected, and OPAT was feasible despite the complexity of these infections. OPAT can avoid multiple and lengthy hospital admissions and maximise time at home and quality of life in this cohort with life-limiting infection.

## Background

Aortic graft infection (AGI) can follow open aortic aneurysmal repair or endovascular aneurysm repair [1]. An estimated 1% of endovascular aneurysm repair (EVAR) devices become infected [2–5]. There is a paucity of published literature to guide management of these complex infections, and most of it focuses on surgical management and outcomes. Surgical explantation followed by appropriate antimicrobial therapy may be curative in select cases, however it is technically difficult, and the role and optimal timing of surgery is unclear [3, 6]. Prolonged and recurrent use of antimicrobial therapy is often required both in patients who undergo surgical intervention and in those who are deemed unsuitable for surgical intervention [7]. Mortality rates are high in both groups [2, 3, 5, 8]. The largest series published to date includes 22 cases of infected aortic endografts [2], and the second most sizeable case series comprises nine cases [9]. Three meta-analyses of aortic graft infections have been published, one of which focuses particularly on infected EVAR [3, 5, 10]. To the best of our knowledge there is no published data focusing specifically on the use of outpatient parenteral antimicrobial therapy (OPAT) for AGI. We examined the presenting features, management, and outcomes of a cohort of patients with AGI following EVAR, with specific focus on the role of OPAT followed by oral suppressive antimicrobial treatment in treating or suppressing these infections.

## Methods

This study was performed in a tertiary referral hospital with a vascular surgery department, an infectious diseases (ID) department and an OPAT programme all on the same site. The majority of the OPAT programme in our hospital is run by the ID department. Joint decisions are made by the vascular surgeons and ID physicians regarding suitability of patients with AGI for OPAT. Once accepted onto the OPAT programme, patients are reviewed by face-to-face weekly visits with an ID physician and OPAT clinical nurse specialist in the ID department. There is a direct telephone line for virtual review of any issues falling outside of weekly visits, and extra in-person review can be organised as needed. Though there is no joint ID/ vascular clinic, review by the vascular surgeons can often be organised on the same or following day if necessary. Standard weekly laboratory testing includes full blood count, renal and liver profiles and C-reactive protein. Once patients complete the course of OPAT and are switched to oral suppressive treatment, the frequency of clinical review is decreased. While on the OPAT programme patients can call the OPAT nurse specialist directly. Once discharged from the OPAT programme patients are followed up in the general ID clinic, which can also be contacted directly if issues arise between visits. Once discharged from care, patients can still phone the ID clinic to be seen if any new clinical issue arises.

The hospital Outpatient Parenteral Antimicrobial Therapy (OPAT) database was used to identify all patients who received at least one course of OPAT for infected EVAR over a 5-year period from January 2014–December 2018. Only AGIs following EVAR were included (referred to here as AGI for brevity). Patient medical and electronic records were accessed to collect data. Management of Aortic Graft Infection Collaboration (MAGIC) case definitions were applied to stratify infections as early or late, suspected or definite [6]. AGI was defined as suspected in a patient with any major criterion, or minor criteria from two of the three categories: clinical/surgical, radiological, or laboratory. AGI was defined as diagnosed in the presence of a single major criterion, plus any other criterion (major or minor) from another category [6]. Patient demographics, clinical features, microbiological findings, management, and outcomes (frequencies and percentages) were described. Descriptive statistical analysis was performed using Microsoft 365 Excel. Patients were followed up until June 2019 for clinical outcomes, and survival was re-assessed in June 2020, which was a median of 36 months (range 25–60 months) after diagnosis of infection for patients still living. British Society of Antimicrobial Chemotherapy standard definitions were used to report patient/infection outcomes and OPAT outcomes [11]. Outcomes were adjudicated retrospectively by the study team based on these guidelines. Patient who completed OPAT and did not require long-term antibiotics were deemed cured. Patients who had partial resolution of infection on OPAT were deemed improved. Patients who had progression or non-response of their infection on OPAT, or who died of any cause, were deemed treatment failure (see Table 3 in Appendix and Table 4 in Appendix for full definitions of OPAT outcomes). No patients were lost to follow up.

## Results

Eleven patients, with a total of 20 OPAT episodes, were identified. Eight patients (72%) met MAGIC criteria for definite infection, and 3 (27%) for suspected infection [6]. Ten of 11 (91%) were male, with a median age of 76 (range 65–85 years, IQR 71–81). The median Charlson co-morbidity index was 6.5. Initial aortic repair was for mycotic aneurysm in one patient, and non-infectious indications in 10/11 patients. Three were emergency procedures, six elective and two semi-elective procedures. Surgical histories prior to presentation with AGI are listed in Table 1. The indication for treatment on OPAT was early AGI (3/11), late AGI (5/11) and mycotic aneurysm (1/11). Three patients commenced OPAT on their first infection episode, and the remaining 8/11 patients had already experienced treatment failure by the time they were commenced on OPAT.

**Table 1:** Clinical features, causative organisms, management and outcomes

Patient No.	Age	Co-mobidity Index	Index device and indication	Early complications/ Index device infection features	Other surgical history prior to Time Zero (first entry to OPAT)	First clinical presentation with infection	Leucocytosis at presentation	Organism(s) cultured	Managment of index device infection	No. of OPAT episodes	Outcome of most recent OPAT episode (months post presentation with infection, cause of death)
1	84	7	Emergency EVAR for AAA Rupture	Bacteraemic at time of EVAR insertion, suspected AEF		Fever	No	*Bacteroides fragilis* (blood culture)	OPAT, lifelong oral suppresion	1	Failure – RIP (10- pancreatic carcinoma)
2	80	4	Elective EVAR for AAA size	Suspected AEF		Fever, abdominal pain	No	*Salmonella enteritidis, Streptococcus anginosis* (blood culture, sac aspirate)	Sac aspirate, OPAT, lifelong oral suppression	3	Partial success, 45
3	79	5	Emergency EVAR for AAA Rupture + fem-fem bypass graft	nNo		Fever, back pain, anorexia, melena	No	None	OPAT, lifelong oral suppression	2	Partial success, 34
4	69	5	Semi-elective EVAR for AAA size + fem-fem bypass graft	Endoleak, LRTI post-op	Repair of endoleak 3 days after initial surgery	Abdominal pain, anorexia	No	None	OPAT, lifelong oral suppression	2	Partial success, 47
5	71	7	Elective EVAR for AAA size	Endoleak	Repositioning of stent	Anorexia, abdominal pain, back pain		*CoNS,* (sac aspirate)	Sac aspirate, OPAT, lifelong oral suppression	1	Failure – RIP (27- severe cardiac failure and cardiogenic shock)
6	81	U	Semi-elective EVAR for AAA size, mycotic aneurysm	Suspected AEF		Back pain	No	None	OPAT, lifelong oral suppression	1	Success, 25
7	68	4	Elective EVAR for AAA size	Fever for 3 days post deployment, no focus found, radiology no collection		Fever	Yes	None	OPAT, oral consolidation 6 months	1	Success, 36
8	75	4	EVAR for AAA size and symptoms	Occlusion of right limb of EVAR, suspected AEF		Fever, abdominal/back pain, anorexia, bleeding	Yes	*Enterococcus faecalis, Bacteroides caccae* (vertebral biopsy)	OPAT, lifelong oral suppression	3	Partial success, 40
9	70	4	Semi-elective EVAR for AAA size and symptoms	Right hip pain	Ax-fem bypass graft right limb balloon angioplasty sac drainage	Anorexia, collection formation	Yes	*Pseudomonas* (sac aspirate, blood culture)	Multiple debridements and drainage, explant + allograft OPAT, lifelong oral suppression	3	Partial success, 25
10	81	7	Elective EVAR for symptoms	Wound infection post-op	Fem-fem bypass graft with development of sinus	Fever, back pain, anorexia, cachexia	No	*MSSA, Pseudomonas aeruginosa, anaerobes, CoNS, Enterococcal sp. (VRE) –* (sinus swabs/excision)	Sinus excision, OPAT, lifelong oral suppression	2	Partial success, 60
11	86	10	Elective EVAR for AAA size	No	Fem-fem cross over for limb occlusion with development of sinus of fem-fem graft	Fever	Yes	*Staphylococcus caprae, candida* (excised sinus)	Sinus excision, OPAT, lifelong oral suppression	1	Failure – RIP (5 – multilobar pneumonia)

*EVAR* Endovascular Aortic Anuerysm Repair, *AAA* Abdominal aortic aneurysm, *Fem-fem* Femorofemoral bypass grafting, *Ax-fem* Axillobifemoral bypass grafting, *MSSA* Methicillin Sensitive *Staphylococcus aureus, CoNS* Coagulase Negative Staphylococcus, *OPAT* Outpatient Parenteral Antimicrobial Therapy, *RIP* Patient dead at time of data analysis

Patients presented with infection a median of 7 months (range 0–81 months) after EVAR. Symptoms at presentation with infection included fever in 6/11(55%), anorexia in 6/11(55%), abdominal pain in 5/11 (45%), back pain in 3/11(27%) and bleeding in 2/11(18%) (Table 1). Only 2 (18%) patients had leucocytosis at the time of presentation. Seven patients had radiological evidence of AGI as per MAGIC criteria; 4 had CT angiograms showing peri-graft fluid; one peri-graft gas; two fat stranding. Two of these patients also had had CT-PET scans demonstrating focally-increased metabolic activity.

Causative organisms were identified in 7/11 (64%) cases, 4 of whom had polymicrobial infections. Three patients (27%) had positive blood cultures, one had positive vertebral biopsy, three a positive culture from sac aspirate and two a positive sinus culture. Five patients had Gram-negative organisms isolated; 3/5 had suspected aorto-enteric fistulae. Organisms isolated included staphylococci and streptococci species(6/11), anaerobes (4/11), enterococci (2/11), *Pseudomonas aeruginosa* (2/11) and *Candida albicans* (2/11) and *Salmonella* species (1/11) (Tables 1 and 2).

**Table 2:** Causative organisms and antimicrobial management on OPAT

Patient No.	Organism(s) Cultured	Antimicrobial therapy 1	Antimicrobial 2 (switch reason)	Antimicrobial 3 (switch reason)	Antimicrobial 4 (switch reason)	Oral suppression
1	*Bacteroides fragilis* (blood culture)	Ertapenem + metronidazole (OPAT, 6 weeks)	PO Moxifloxacin (oral consolidation)			Co-trimoxazole, RIP on suppression
2	*Salmonella enteritidis, Streptococcus anginosis* (blood culture, sac aspirate)	Ceftriaxone, 6 weeks	PO Co-trimoxazole (oral consolidation)			Co-trimoxazole
3	None	Ertapenem+ daptomycin, 6 weeks	PO cefuroxime (oral consolidation)			Co-trimoxazole
4	None	Ertapenem + daptomycin, 8 weeks	PO Linezolid + cefixime (oral consolidation)	Ertapenem and daptomycin (rising inflam markers)	PO Linezolid + cefuroxime	Cefuroxime
5	*CoNS,* (sac aspirate)	Piperacillin- Tazobactam + daptomycin	Teicoplanin +linezolid (admitted for graft reposition, unclear reason for antibiotic change)	Piperacillin- Tazobactam + daptomycin, 6 weeks	PO Ciprofloxacin +metronidazole (oral consolidation)	Co-trimoxazole, RIP on suppression
6	None	Co- Amoxiclav IV, 6 weeks	Cefuroxime PO (oral consolidation)			Amoxicillin
7	None	Co-amoxiclav IV, 6 weeks	Co-amoxiclav PO (oral consolidation, 6 months)			None
8	*Enterococcus faecalis, Bacteroides caccae* (vertebral biopsy)	Ertapenem+metronidazole, 8 weeks	Meropenem^a^ + caspofungin^a^ + PO linezolid (deterioration and pain)	Meropenem^a^, PO fluconazole (anaemia on linezolid and caspofungin)		Co-amoxiclav + Fluconazole
9	*Pseudomonas* (sac aspirate, blood culture)	Piperacillin-tazobactam + IV/PO ciprofloxacin on and off × 1 year, on OPAT between multiple temporising measures	Piperacillin-tazobactam + daptomycin (added for pneumonia)	Vancomycin (admitted with pneumonitis on daptomycin) + meropenem (later rash on piperacillin-tazobactam)	PO Ciprofloxacin + PO co-amoxiclav (oral suppression)	Ciprofloxacin + co-amoxiclav
10	*MSSA, Pseudomonas aeruginosa, anaerobes, CoNS, Enterococcal sp. (VRE) –* (sinus swabs/excision)	Piperacillin-tazobactam + PO rifampicin, 12 weeks	PO Ciprofloxacin (oral consolidation, 1 year)	Meropenem + daptomycin (re-treatment 2 years later)	Ertapenem + daptomycin (OPAT, 6 months)	Ciprofloxacin
11	*Staphylococcus caprae, candida* (excised sinus)	Piperacillin-tazobactam, 12 weeks	PO co-amoxiclav (oral consolidation)	Daptomycin (admitted with pneumonia)	Vancomycin (eosinophilia on daptomycin)	RIP 5 months, inpatient on IV antibiotics for pneumonia

*IV* Intravenous, *PO* Oral

^a^self-compounded and administered

Twenty OPAT episodes were analysed, with patients having a median of two (range 1–3) episodes. Most patients (10/11) received more than one intravenous antimicrobial agent at some stage during the course of their treatment, including their inpatient stay. Six patients received dual IV antimicrobial therapy on OPAT. All patients received oral antibiotics for a minimum duration of 6 months after OPAT and some received concomitant oral antibiotics during their OPAT treatment. Antibiotics received on OPAT can be found in Table 2. A single patient stopped oral antibiotics and is alive at 36 months after diagnosis of infection. This patient had an early suspected infection (two minor MAGIC criteria) following elective EVAR for AAA size. The patient received 12 weeks of empiric antimicrobials, followed by 6 months of oral suppressive therapy (Patient 7, Tables 1 and 2). All other patients were continued on life-long oral antibiotics – the three patients who reached primary endpoint of death were still on antibiotics at the time of death, and the seven surviving patients were still on oral suppressive therapy at the time of data collection. Five patients had temporising measures as part of their infection management (Table 1). Only one patient underwent surgical explantation of the EVAR graft (Patient 9).

Three patients received more than two OPAT courses to treat clinical flares of infection. Patient 9 had recurrent abscess formation at the site of axillobifemoral graft; this patient had multiple temporising measures (sac drainage) and three OPAT episodes, before eventually having elective graft explantation. The patient had a stormy post-operative course with a long admission to intensive care but was alive and on oral antimicrobials 25 months after diagnosis of AGI and 15 months after surgical explantation of the graft. Patient 8 successfully completed 12 weeks of OPAT, and later presented with fatigue and rising inflammatory markers while on oral suppression therapy; the patient was switched back to intravenous antibiotics until symptoms and markers improved. This was done on two occasions and on one occasion was achieved without a hospital admission. Patient 4 had increasing abdominal pain and fevers on oral suppressive therapy and was admitted for switch to intravenous (IV) antibiotics and repeat surgical opinion. The decision was made that the patient was still not for surgical intervention and he was discharged within a week via OPAT. At the time of writing, he was on oral suppressive therapy, clinically stable, but with deteriorating radiological appearances.

A total of five adverse events occurred in three patients during OPAT; all were antimicrobial-related. One patient presented with a daptomycin-induced eosinophilic pneumonitis for which they required oxygen therapy and antibiotics were changed to vancomycin (Patient 9). He made a full recovery from the pneumonitis. A second patient was admitted with transfusion-requiring anaemia during therapy with linezolid and caspofungin (Patient 8). Three other adverse events occurred which did not require re-admission; piperacillin/tazobactam-induced skin rash, cefuroxime-induced skin rash, and asymptomatic eosinophilia on daptomycin.

Four patients had to be re-admitted for infection-related reasons at least once while on OPAT; patient 9 was admitted 3 times (twice for failure of therapy and once for complications of antibiotics, as described above); patient 8 was admitted twice (once for failure of therapy and once for complications of antibiotics). Patient 4 was re-admitted for failure of therapy and a repeat surgical opinion and patient 7 was re-admitted electively for repeat imaging (Tables 1 and 2).

Eight (72%) patients were alive at a median of 36 months of follow up (range 25–64 months) (Table 1). OPAT outcomes for the 20 episodes were – success (9/20), partial success (3/20), failure (5/20), and indeterminate (3/20). Patient/ infection outcomes for the 11 patients were – cure (1/11), improved (7/11), failure (3/11) (see Table 3 in Appendix and Table 4 in Appendix for definitions of OPAT outcomes). The patient deemed cured had clinical and radiographical improvement on OPAT and was off all antibiotics. In the improved group, three patients had stable radiological findings and were clinically well and four had signs of disease progression radiographically but were stable clinically. All in this group were currently at home on oral antibiotics. Three patients died at 5, 10, and 27 months, of cardiogenic shock, pancreatic carcinoma and multi-lobar pneumonia respectively (treatment failure according to guidelines), however no deaths attributable to vascular graft infection occurred during the follow-up period. The patients who died were older (median age 84 vs 76 years) and had more co-morbidities (Charlson co-morbidity index median 4 vs 6.5), as compared with survivors, although numbers were too small to perform any statistical test for differences Two were still on suppressive oral antibiotics at the time, and one was on intravenous antibiotics for pneumonia. Of the five patients who had no surgical intervention at all, 4/5 were still alive at 36–47 months post presentation. Palliative care referral was offered to 2 of the 3 patients who died. One of the 8 surviving patients declined referral to palliative care services.

The median number of days on OPAT was 46.5 (range 14–91 days). Patients were on IV antibiotics for a total of 1560 days; 696 in hospital and 864 on OPAT. OPAT facilitated a saving of 864 total hospital days, representing a 55% reduction in length of stay.

## Discussion

Our patients had similar demographics to those in previously published literature on aortic vascular endograft infections, insofar as they were mostly male, older and with multiple co-morbidities, and these latter factors correlated with mortality [2, 5, 9, 10]. The median time to presentation with infection in our cohort was similar to that reported previously [2]. Fever, pain and anorexia were common presenting features in our data and have been previously reported [2, 9]. Notably, most of our patients had a normal white blood cell count at presentation with infection, in contrast to prior cohorts [2]. Where infecting organisms are identified for AGIs, they are predominantly endogenous, and include a broad range of species [10, 12–14]. The incidence of polymicrobial infection was notable here, in line with other series [2, 15]. The potential for more than one culprit organism should be considered when treating empirically. A relatively high proportion (4/11, 36%) had suspected aorto-enteric fistulae [2, 10]. Fistulae should be suspected in the case of polymicrobial infections, especially with Gram negative or fungal organisms. A significant proportion of patients had negative cultures, in agreement with previously described patients, possibly related to prior antimicrobial use [2, 8–10, 12]. This complicates the selection of antibiotics, leads to the use of more broad-spectrum antibiotics and/or multiple antibiotics, increasing the chances of antibiotic side effects or adverse events. This needs to be taken into consideration when assessing patients’ suitability for treatment on OPAT. It does not, however, preclude the use of OPAT; all four patients with no guiding microbiology were successfully treated.

Expert consensus favours surgical removal of an infected graft where possible [2, 8]. One of the most notable findings here was the longer-than-expected survival, especially given the low rate of surgical intervention. In our series 8/11 (72%) of all patients (including suspected cases) and 5/8 (62%) of definite cases were alive at a minimum of 25 months (median 36 months) of follow up post-diagnosis of infection, despite only one of these patients having undergone graft explantation. In the published literature, despite a high (~ 30%) peri-operative mortality rate, patients who have undergone radical surgical intervention have survived longer than those who have not [2, 5, 6, 10]. Indeed, in one case series, all patients who did not undergo explantation had died within 2 years of presentation with infection [2]. A review of published literature looking specifically at non-explanted AGI showed slightly increased survival in those who had some sort of temporising procedure (drainage, surgical debridement, sac irrigation, and/or omentoplasty) as well as antimicrobial therapy, as compared with antimicrobial therapy alone (59% vs 50%) [14]. Our overall (27%) and medically managed (20%) all-cause mortality compares favourably, and there was no infection-attributable mortality despite less use of temporizing or definitive procedures.

However, this small selective cohort cannot be used to compare surgical versus medical therapy for AGI, but only to describe outcomes in those who survive and are considered candidates for outpatient management. There is an inherent bias in the selection of patients for analysis from an OPAT registry, as those who presented in extremis or died during the first presentation of infection are not included. By definition, the patients in our cohort were well enough for hospital discharge at least once with OPAT, skewing overall survival as compared with cohorts including all patients who present with AGI. In a series including all presenting AGI patients, 17/22 (77%) presented with an urgent surgical indication (rupture or bleeding), as compared with only two of our patients presenting with bleeding and none with rupture [2]. Perhaps these emergent events represented a late presentation of infection, and our cohort may have been at an earlier point in AGI, more amenable to medical therapy. Co-morbidity indices have not consistently been reported for comparison and could be useful for stratifying risks. Overall, however, we think it is unlikely that our patients were a much fitter cohort than those previously described; they were elderly with multiple co-morbidities, and potentially less fit for surgery. A substantial proportion also had presumed aorto-enteric fistulae, a recognised risk factor for poor outcome. In addition, the experience of individual surgical centres with, and skill in, performing primary explantation and in-situ allo/autograft reconstruction may also impact decisions around intervention in those who would elsewhere be considered for radical surgery. Finally, mortality is a crude outcome measure. Patients who survive explant and reconstruction may return to normal function and activity, whereas some survivors in the medically managed group may continue to decondition in the setting of chronic infection. Such qualitative outcomes should be measured to add more meaningful outcome data to decisions about the best course of action.

To the best of our knowledge there is no other published data on OPAT outcomes for AGI. As with other complex infections, OPAT can deliver significant savings in inpatient bed-days and healthcare costs [10]. This study did not involve a formal cost analysis, but with more than half of the total intravenous antibiotics for these infections being delivered in the outpatient setting, it is likely that the use of OPAT incurred cost savings to the healthcare system. Delivering antibiotics in the outpatient setting also has psychological and emotional benefits for patients and their families, especially those for whom this is a life-limiting condition [11]. The nature of AGIs necessitates regular ongoing assessment of response to antimicrobial therapy, whether intravenous or oral, and the goals of therapy should be made clear to patients. Proposed changes to British Society for Antimicrobial Chemotherapy (BSAC) OPAT outcome measures suggest starting with defining a treatment goal (cure, improvement, palliation) and then assigning an OPAT outcome (aim attained, aim not attained, indeterminate, death) [11]. These proposed changes would improve multi-disciplinary planning and patient involvement in shared decision-making in complex infections such as AGI. Adverse events and need for readmission in this cohort were picked up with standard weekly face-to-face outpatient review by an ID physician, demonstrating appropriate safety-netting. While no deaths attributable to vascular graft infection occurred during the follow-up period in our series, AGI carries a high mortality rate as described above and this should be addressed early in the treatment course. OPAT has been used indefinitely in certain palliative situations, and some patients with AGI may be appropriate candidates for this approach [16, 17]. Discussions about re-admission and preparedness for potential vascular catastrophes in the community may also be appropriate for certain patients, and palliative care referral should be offered where appropriate.

## Conclusion

OPAT has multiple roles in the setting of AGI, and both patients who undergo surgery and those who do not may benefit. OPAT may be used as a temporizing measure in those awaiting radical surgery, as induction therapy in those AGIs for medical management, and for treatment of clinical ‘flares’ in patients otherwise well-maintained on oral suppressive therapy. In each of these instances, OPAT can avoid multiple and lengthy hospital admission, reduce costs and maximise time at home and quality of life in this cohort with life-limiting infection. Importantly, we have also demonstrated that long-term outpatient medical management (OPAT followed by oral suppressive therapy) of patients with AGI for whom cure is not intended is feasible. No deaths attributable to vascular graft infection occurred during the follow-up period, but the prognosis and unpredictability of AGI warrants early setting of treatment goals by a multi-disciplinary team comprised of vascular surgeons, infections specialists and, where appropriate, palliative care practitioners. Patients with early AGI and frailty who are not considered candidates for curative surgery should be counselled about their prognosis and consider criteria forre-admission and preparedness for potential vascular catastrophes in the community if appropriate. OPAT followed by oral suppressive therapy can be a suitable long-term non-curative treatment option for certain patients with AGI.

## Data Availability

The datasets generated during this study are not publicly available due to patient identifier information but can be made available from the corresponding author on reasonable request.

## Appendix 1

**Table 3:** Definition of OPAT Outcomes, The British Society for Antimicrobial Chemotherapy Outpatient Parenteral Antimicrobial Therapy National Outcomes Registry System (NORS) User Guide, Section 3.8, Table 1 Patient Outcomes [11]

Infection Outcomes	
Cure	Completed OPAT therapy +/− oral step down for defined duration with resolution of infection and no requirement for long term antibiotic therapy
Improved	i. Completed OPAT therapy +/− oral step down with partial resolution of infection but need for further follow up OR ii. Completed OPAT therapy but required escalation of antimicrobial therapy during OPAT (without admission) +/− oral step down with ultimate cure or partial improvement (as above)
Failure	Progression or non-response of infection despite OPAT, required admission, surgical intervention or died for any reason

## Appendix 2

**Table 4:** Definition of OPAT Outcomes, The British Society for Antimicrobial Chemotherapy Outpatient Parenteral Antimicrobial Therapy National Outcomes Registry System (NORS) User Guide, Section 3.8, Table 2 OPAT Outcomes [11]

OPAT Outcomes	
Success	Completed therapy in OPAT with no change in antimicrobial agent, no adverse events, cure or improvement of infection and no readmission
Partial Success	Completed therapy in OPAT with either change in antimicrobial agent or adverse event not requiring admission
Failure of OPAT	Readmitted due to infection worsening or due to adverse event. Death due to any cause during OPAT
Indeterminate	Readmission due to unrelated event e.g. chest pain
